# Associations between Power Training-Induced Changes in Body Composition and Physical Function in Older Men: A Pre-Test–Post-Test Experimental Study

**DOI:** 10.3390/ijerph20227070

**Published:** 2023-11-16

**Authors:** Sindre H. Fosstveit, Kolbjørn Lindberg, Thomas Bjørnsen, Erlend E. Sibayan, Joachim S. Fjeller, Sondre Løvold, Tommy Kolnes, Fredrik T. Vårvik, Sveinung Berntsen, Hilde Lohne-Seiler

**Affiliations:** Department of Sport Science and Physical Education, Faculty of Health and Sport Sciences, University of Agder, 4604 Kristiansand, Norway; kolbjorn.a.lindberg@uia.no (K.L.); thomas.bjornsen@uia.no (T.B.); erlendos.sibayan@live.no (E.E.S.); joachim.fjeller@gmail.com (J.S.F.); sondrlov@gmail.com (S.L.); t_m_k_92@hotmail.com (T.K.); fredrik.t.varvik@uia.no (F.T.V.); sveinung.berntsen@uia.no (S.B.); hilde.l.seiler@uia.no (H.L.-S.)

**Keywords:** ageing, body fat, disability, exercise, public health, sarcopenia

## Abstract

Background: It is well-established that cross-sectional measurements of poor body composition are associated with impaired physical function and that power training effectively enhances total lean mass and physical function in older adults. However, it is unclear if power training-induced changes in body composition are associated with improved physical function in older adults. Aim: The present study investigated associations between body composition and physical function cross-sectionally and with power training-induced changes in older men. Methods: Forty-nine older men (68 ± 5 yrs) completed a 10-week biweekly power training intervention. Body composition was measured using dual-energy X-ray absorptiometry. Physical function was assessed as a composite Z-score combining measures from Sit-to-stand power, Timed up-and-go time, and loaded and unloaded Stair-climbing time (15 steps). Linear and quadratic regression analyses were performed to assess associations between body composition and physical function. Results: At baseline, total (R^2^ = 0.11, *p* < 0.05) and percentage body fat (R^2^ = 0.15, *p* < 0.05) showed a non-linear relationship with physical function. The apex of the quadratic regression for body composition was 21.5% body fat. Furthermore, there was a non-linear relationship between changes in body fat percentage and physical function from pre- to post-intervention (R^2^ = 0.15, *p* < 0.05). Conclusion: The present study’s findings indicate that participants with a body composition of ~20% body fat displayed the highest level of physical function at baseline. Furthermore, despite small pre–post changes in body fat, the results indicate that those who either preserved their body fat percentage or experienced minor alterations observed the greatest improvements in physical function.

## 1. Introduction

The global older population is rapidly growing, and by 2050, over two billion will be over the age of 60, more than double the amount since 2015 [1]. A degenerative loss of muscle mass (sarcopenia), accompanied by reduced muscle strength and power, will induce a critical loss of physical function for many at this age [2]. Physical function is defined as the capacity to perform essential physical actions necessary for independence in daily living, as well as discretionary activities that can enhance the quality of life [3]. Reductions in physical function can result in a decrease in the ability to live independently, which, from a public health perspective, could be one of the most considerable challenges of this century [4,5].

Moreover, declines in physical function due to ageing and changes in body composition co-occur [6]. For instance, excessive body fat accumulation represents additional body mass that provides no additional contribution to movement performance and potentially restricts physical function due to the energy cost of moving the enhanced load [7,8]. Nonetheless, there are conflicting results with respect to whether the decrease in lean mass or increase in body fat is associated with impaired physical function. Despite the fact that some cross-sectional studies have reported a positive association between lean mass and physical function in older adults [9,10], other cross-sectional studies have reported no association between lean mass and physical function but a clear negative association between body fat and physical function [7,11,12,13]. However, most studies have concentrated on overweight/obese individuals and have not investigated potential non-linear relationships [9,10,11,12,13]. Notably, a study by Hardy et al. [14] demonstrated a non-linear relationship between body composition (estimated using Body Mass Index (BMI)) and physical function in older adults, indicating that neither excessive nor too-low body fat levels are beneficial for physical function.

The association between body composition and physical function is multifaceted, with low and high body fat percentages potentially negatively impacting physical function. Low body fat percentages are associated with poor health, frailty, osteoporosis, and decreased muscle strength [6,15,16], potentially leading to compromised knee and hip joint functions essential for maintaining balance [17]. On the other hand, high body fat percentages can result in destabilised postures, contributing to static and dynamic stability difficulties, and are associated with musculoskeletal conditions that further impair physical function [18,19].

Several exercise interventions have been suggested to counteract age-related declines in physical performance and body composition and their subsequent impacts on physical function [20,21,22,23,24]. Among these interventions, power training has gained increasing attention due to its multifaceted advantages. Power training focuses on enhancing neuromuscular power, which is defined as the product of force and distance over time (force × distance/time). In older adults, neuromuscular power is a superior predictor of functional status compared with maximal muscle strength or muscle mass [20]. The ability to execute rapid movements in daily tasks, like swiftly crossing a road or regaining balance after tripping, is more crucial than maximal strength [25]. The National Strength and Conditioning Association has underscored the significance of power training for older adults’ physical function, recommending generic power training programmes [21]. A power training programme that combines both heavy and low loads may be most advantageous, as it elicits force- and velocity-related neuromuscular adaptations, leading to improved physical function [26]. Notably, several recent studies have shown that power training interventions can additionally enhance total lean mass and muscle size in adults [27] and older adults [28]. In light of the existing research, there is a growing interest in comprehending the underlying causes for the enhancements in physical function due to power training and, specifically, the potential power training-induced changes in body composition. It is well-established that cross-sectional measurements of poor body composition are associated with impaired physical function and that power training can prevent and counteract the negative age-related declines in body composition and physical function [6,14,20,21,28,29]. However, individual differences in body composition and physical function at cross-sectional investigations are considerably larger than within-subject training-induced changes. Thus, it is unclear if power training-induced changes in body composition are associated with improved physical function in older adults.

Therefore, the aim of the present study was to investigate the associations between body composition and physical function cross-sectionally and with power training-induced changes in these variables in older men (60–80 years). We hypothesised that the body composition of older men would follow a non-linear relationship with physical function.

## 2. Materials and Methods

### 2.1. Study Design

**Figure 1:**
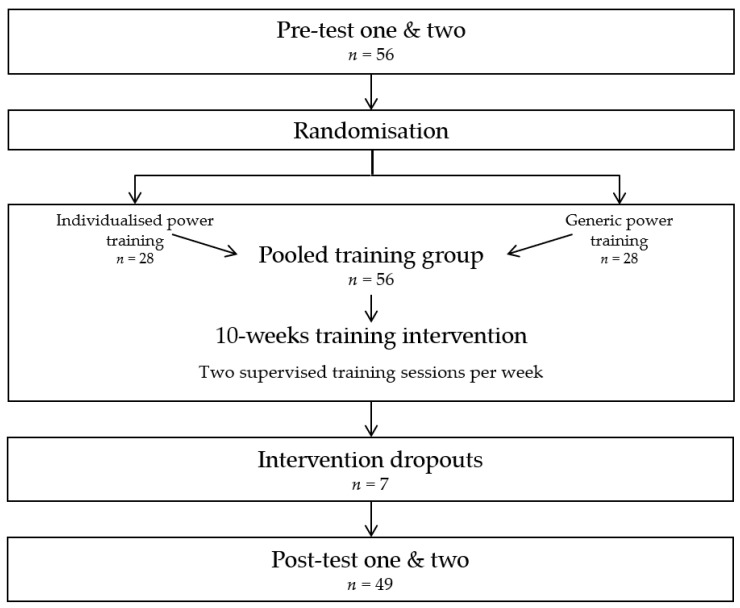
Study design flowchart illustrating the timeline for testing, randomisation, intervention, and intervention dropouts. Arrows indicate the progression of participants from initial testing through randomisation into individualised or generic power training groups, their subsequent consolidation into a pooled training group for the intervention, and finally to post-intervention testing.

A more detailed description of methods and training group comparisons from the randomised controlled trial was previously published in Lindberg et al. [26]. Briefly, participants were randomly assigned to either a generic power training group or an individualised power training group based on force–velocity profiles. However, both exercise groups aimed to improve strength, power, physical function, and body composition. In the present study, the training groups were pooled to assess associations between body composition and physical function (Figure 1). All participants were familiarised with testing procedures and training protocols to minimise potential learning effects. Thereafter, participants performed two pre- and two post-intervention test sessions before and after a 10-week biweekly training period (Figure 1). The same standardised protocol was performed under both pre-and post-testing to ensure that the protocol was identical for each participant.

### 2.2. Study Participants

A total of 56 voluntary participants (age 68 ± 5 yrs; height 179 ± 7 cm; weight 83 ± 10 kg) were recruited from Kristiansand, Norway. Target participants were healthy home-dwelling men who were recruited with advertisements in the local newspaper. Participants were also invited to an information meeting and screened for study inclusion. Inclusion criteria were as follows: male, aged 60–80 years, healthy (not having cognitive impairment, acute or terminal illness, or severe cardiovascular, respiratory, musculoskeletal, or neurological diseases disturbing voluntary movement), and able to participate in heavy and explosive strength training. Exclusion criteria were any illness or disease that prevented them from safely participating in resistance exercise, and they could not have participated in systematic resistance exercise (≥one session per week) six months prior to study entry. All participants received oral and written information about this study, signed an informed consent form to participate, and had to obtain a written health certificate from their medical doctor. During the intervention, participants could not perform any other form of resistance or strenuous exercise. Seven participants could not complete the intervention due to reasons unrelated to the study, while 49 participants completed the entire study and were included in the analyses.

### 2.3. Study Intervention

All 49 participants completed a 10-week biweekly supervised power training intervention. The first two training sessions were conducted submaximal (lower weights and movement velocity) to familiarise participants with the training protocol and ensure proper technique and execution. All training sessions were separated with a minimum of 48 h of rest. Both the individualised power training group and the generic power training group trained with an equal combination of low-load (<50% of one-repetition maximum (1 RM)) high-velocity strength training and heavy-load (>70% of 1 RM) strength training on average, where 1 RM refers to the maximum amount of load an individual can lift for one repetition of a specific exercise. The only difference between groups was that the individualised power training group had their training focus divided based on a median split of the participant’s force–velocity slope, as described in Lindberg et al. [26]. The participants were instructed to perform both the high- and low-load exercises with maximal intentional velocity. The loading of each training exercise was based on repetitions in reserve [30]. Subject participation was recorded at every training session, and those with less than 80% attendance were excluded from this study. Each session lasted one hour and started with a general 5–10-min warm-up held by the present instructor. The warm-up included light aerobic movements (i.e., walking on a treadmill, cycling, or stair climbing) and light dynamic stretching.

### 2.4. Study Outcomes

The present study included the Sit-to-stand test, Timed-up-and-go test, and Stair-climbing test to measure physical function, while dual-energy X-ray absorptiometry (DXA) was included to measure body composition.

The Sit-to-stand power test was based on principles by Lohne-Seiler, et al. [31]. The test was performed on a force platform (MuscleLab; Ergotest, Langesund, Norway) connected to an integrated data analysis program (MuscleLab; Ergotest, Langesund, Norway). After a given signal, the participants were encouraged to stand up as fast as possible and jump (if possible) from a chair without handrails (height 46.0 cm). The participants performed two attempts per load with a rest interval of 2 min between loads. A total of four loads from bodyweight to an effort with a heavyweight were utilised. The load was applied using both weight vests and dumbbells. All attempts were recorded, and the best valid effort was used. Test–retest reliability of peak neuromuscular power revealed a coefficient of variation (CV%) of 4.4 and interclass correlation (ICC) of 0.95, and paired *t*-tests established no significant differences (*p* > 0.05) between pre-tests 1 and 2.

The Timed up-and-go test was performed in accordance with Schoene et al. [32]. In this test, the time an individual needs to rise from a standard armchair (seat 46 cm high) based on a signal from the test leader, walk 3 m, turn around, return to the chair, and sit down again is measured. Participants were instructed to walk as fast as possible without running. If a participant failed to follow the instructions, the attempt was not registered and had to be performed again. The test was performed two times as quickly as possible and measured using a stopwatch, where the fastest time recorded in seconds was used for analysis. The test–retest reliability of the Timed up-and-go test revealed a CV% of 4.5 and ICC of 0.71. Paired *t*-tests established no significant differences (*p* > 0.05) between pre-tests 1 and 2.

The Stair-climbing test was based on a previous study performed by Walker et al. [33]. Participants were instructed to climb 15 steps (16 cm per step) as fast as possible. The time was recorded using photocells (Brower Timing Systems, Draper, UT 84020, USA) placed at the bottom and top of the stairs at 85 cm height. Before the test started, participants were instructed to walk fast up and down the stairs as a warm-up. After the warm-up, participants made two unloaded attempts and two with a 20 kg vest (Sportsmaster, product number: ASL503). All attempts were recorded in both Stair-climbing tests (unloaded and loaded). The results from the two tests were analysed separately, and the best attempt was used for analysis. The test–retest reliability was examined between pre-tests 1 and 2 for unloaded (CV% = 6.7; ICC = 0.76) and loaded (CV% = 3.5; ICC = 0.90) Stair climbing tests. Paired *t*-tests established no significant differences (*p* > 0.05) between consecutive tests.

Body composition was determined with DXA using a Lunar Prodigy (model 8743; GE Lunar Corporation, Madison, WI, USA). Participants were asked not to engage in strenuous physical activity 24 h before the measurements. The DXA measurements were taken after overnight fasting to ensure minimal variations in hydration and food intake [34]. All participants were scanned in the standard mode automatically chosen by the machine. Following the manufacturer’s guidelines, the machine was calibrated daily every morning prior to any assessments. Images were analysed with encore software (version 14.10.022; GE-Healthcare). Body mass was divided into bone minerals and soft tissue from the X-ray scan, where the soft tissue was divided into body fat and lean mass [35]. DXA reliability was not measured in the present study. However, previous test–retest analyses of 31 older men from our lab [36] demonstrated an intraclass correlation of 0.99 (*p* < 0.001) and CV of 1.5% for total lean mass, 3.8% for total fat mass, and 3.9% for body fat percentage.

### 2.5. Ethics Approval and Consent to Participate

This study was approved by the ethical board of the University of Agder (Faculty of Health and Sport Sciences) and the Norwegian Centre for Research Data (ref. number: 923574) and performed in agreement with the Declaration of Helsinki. All participants received oral and written information about this study and signed a consent form to participate.

### 2.6. Statistical Analyses

Analyses were performed using MATLAB R2021a (Mathworks, Natick, MA, USA) and IBM SPSS Statistics 28 for Windows (v. 28; IMB Corp., Armonk, NY, USA). The average results from the two baselines and two post-tests were used in the analyses. Physical function was assessed as a composite Z-score combining measures from Sit-to-stand power, Timed-up-and-go time, and loaded and unloaded Stair-climbing time. Linear and quadratic regression analyses were performed to determine associations between body composition and physical function, while paired samples t-tests were performed to determine pre- to post-changes. The quadratic regression analyses were included to assess the linearity of associations. All 49 participants performed pre- and post-tests in all measurements, except for two in the Sit-to-stand test due to illness, five in the loaded Stair climbing due to back and knee pain, and one in DXA measurements for personal reasons. The value of *p* < 0.05 was considered statistically significant in all analyses. The test–retest reliability was examined using the coefficient of variation and interclass correlation for each variable and was utilised by using a freely accessible spreadsheet [37] (http://www.sportsci.org/resource/stats/index.html, accessed on 21 May 2022). Consecutive pairwise comparisons were examined, i.e., test 1 versus test 2, to determine any systematic differences between the trials’ reliability statistics.

## 3. Results

### 3.1. Study Participants

The main characteristics at baseline for the participants, as well as body composition and physical function changes from baseline, are shown in Table 1. Total lean mass and body fat percentage significantly changed during the intervention (1.3 ± 2.0%, *p* < 0.05; −1.2 ± 5.7%, *p* < 0.05, respectively).

### 3.2. Cross-Sectional Associations—Body Composition and Physical Function

**Figure 2:**
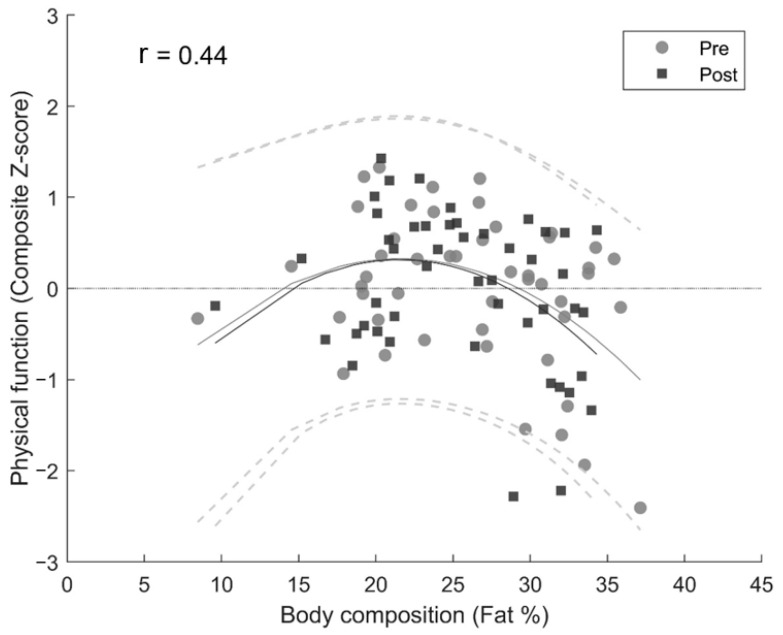
Cross-sectional associations between body composition and physical function. The solid lines represent mean values, and the dashed lines represent values of the 95% CI.

At baseline, total lean mass was positively associated with Sit-to-stand power (R^2^ = 0.32, B = 34.7 [19.3; 50.1], *p* < 0.05) but not with physical function as a composite Z-score measure (*p* > 0.05). Total body fat (R^2^ = 0.11, F(2, 45) = 3.92, *p* < 0.05) and body fat percentage (R^2^ = 0.15, F(2, 45) = 5.26, *p* < 0.05) had a significant non-linear relationship with physical function (Figure 2). The apex of the quadratic regression for body composition was 21.5% body fat.

### 3.3. Power Training-Induced Changes in Body Composition and Physical Function

**Figure 3:**
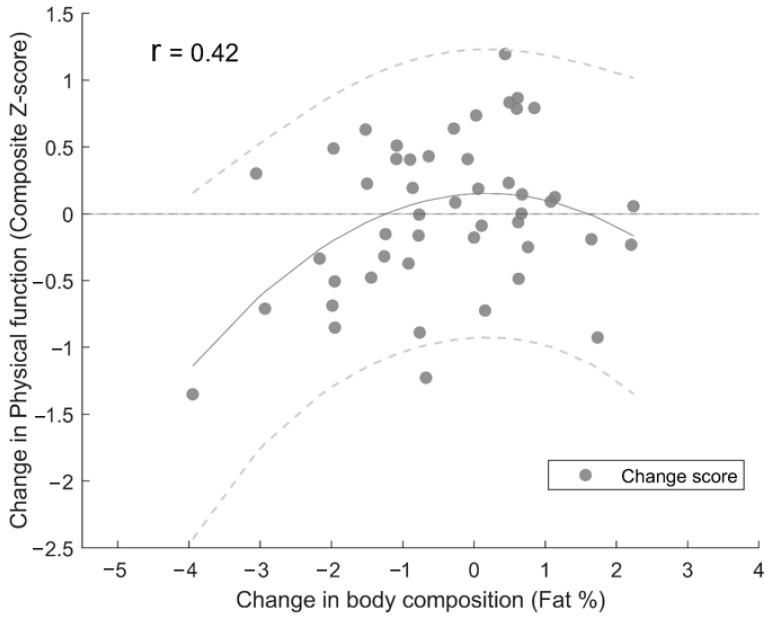
Associations between power training-induced changes in body composition and physical function. The solid line represents the mean value, and the dashed lines represent values of the 95% CI.

Total lean mass (1.3 ± 2.0%, *p* < 0.05), body fat percentage (−1.2 ± 5.7%, *p* < 0.05), Timed-up-and-go time (−1.6 ± 4.9%, *p* < 0.05), unloaded Stair-climbing time (−5.2 ± 6.7%, *p* < 0.05), and loaded Stair-climbing time (−4.1 ± 6.8%, *p* < 0.05) all improved during the power training intervention (Table 1). Furthermore, there was a significant non-linear relationship between changes in body fat percentage and physical function from pre- to post-intervention (R^2^ = 0.15, F(2, 45) = 4.94, *p* < 0.05) (Figure 3).

## 4. Discussion

The present study aimed to investigate the associations between body composition and physical function cross-sectionally and with power training-induced changes in these variables in older men.

The main findings showed that total body fat and body fat percentage had a significant non-linear relationship with physical function at baseline. In the present study, participants with a body composition of ~20% body fat displayed the highest level of physical function. Furthermore, total lean mass, body fat percentage, Timed-up-and-go time, and unloaded and loaded Stair-climbing time improved during the 10-week power training intervention. Additionally, there was a significant non-linear relationship between power training-induced changes in body fat percentage and physical function pre- to post-intervention. The participants who either preserved their body fat percentage or experienced minor alterations appeared to obtain the greatest improvements in physical function pre- to post-intervention.

The present study’s findings at baseline are in accordance with recent cross-sectional studies supporting the association between body composition and physical function in older men [7,11,12,13,14]. At baseline, total lean mass was positively associated with Sit-to-stand power but not physical function as a composite Z-score measure. These results are supported by Orssatto, Bezerra, Schoenfeld and Diefenthaeler [7], Bouchard, Beliaeff, Dionne and Brochu [11], Woo, Leung and Kwok [12] and Jankowski, Gozansky, Van Pelt, Schenkman, Wolfe, Schwartz and Kohrt [13], who, respectively, reported no association between lean mass and physical function. Although some studies [9,10] reported a positive association between lean mass and physical function in older adults, there are conflicting results. As for the results in the present study, it may seem like lean mass is not associated with measures of physical function that involve gait speed (Timed-up-and-go and Stair-climbing tests) but with measures of jumping power (Sit-to-stand test). The findings agree with a study by Buehring et al. [39], who discovered no association between lean mass and physical function measures included in the Short Physical Performance Battery (balance, gait speed, and timed chair rise) but found an association between lean mass and jumping power. One explanation may be due to the fact that walking, agility, and balance are affected by several non-morphological factors, such as the sensory systems and sensory–motor integration [40,41].

Moreover, another explanation for the positive association between lean mass and jumping power may be due to the additional strength demand of the Sit-to-stand test compared with the Stair-climbing and Timed-up-and-go tests. Lean mass is strongly related to maximal strength development [42], and maximal strength is an essential part of maximal power performance (product of force and distance over time) [20]. Hence, it is likely that the participants with a higher amount of lean mass would exert higher power.

Furthermore, the present study discovered a non-linear relationship between body fat percentage and physical function at baseline, indicating that neither excessive nor too-low body fat levels may benefit physical function in older men. The potential non-linear relationship may be of particular interest as it could help identify an optimal body fat percentage threshold for optimising older men’s physical function. In the present study, participants with a body composition of ~20% body fat displayed the highest level of physical function. Additionally, impaired physical function was primarily observed in participants with the highest body fat percentage (⪆30% body fat) but with an indication of impaired physical function in participants with the lowest body fat percentage (⪅15% body fat). However, stratified analyses could not be conducted due to a limited number of underweight individuals. The findings agree with Hardy et al. [14], who discovered a non-linear relationship between body composition (estimated using BMI) and physical function (chair rise, walking speed, and standing balance tests) in older men and women. Furthermore, as for the results from the present study, Hardy et al. [14] discovered that impaired physical function was primarily observed in participants with the highest BMI but with some indication of impaired physical function also in the participants with the lowest BMI. It is noteworthy that BMI is an inaccurate measure of body fat percentage on an individual level; however, the measurements correspond relatively well within groups and distinct categories of body fatness at the group level [43].

Impaired physical function among the participants with the lowest body fat percentage may be due to a plethora of variables. Previous research has highlighted several factors, such as poor health, low levels of physical activity and frailty, all of which may influence physical function [6,15]. Being underweight is a risk factor for osteoporosis, decreased muscle strength, and a weakened immune system [16]. Such conditions can lead to slower step responses, an impaired capacity of the knee joint to absorb impact forces, and a reduced ability of the hip joint to generate the power necessary to control trunk movement during balance–recovery steps [17]. Moreover, considering the high number of healthy-weight participants (*n* = 18) with a relatively high functional ability level at baseline (Timed up-and-go time = 4.1 ± 0.3 s) accompanied by no underweight participants, any such relationship may be weakened. The association between impaired physical function and low body fat percentage may arguably have been stronger with a higher number of underweight participants.

Physical function impairment in participants with the highest body fat percentages may also be attributed to specific biomechanical and physiological factors. Notably, obesity often results in a forward shift of the body’s centre of mass, affecting posture during both standing and walking activities, thereby compromising static and dynamic stability [18,19]. Furthermore, obesity is linked to various musculoskeletal conditions that can impact bodily movement and postural balance, resulting in decreased physical function and an increased tendency for falls [18,19]. Older adults generally perform their daily living activities near maximum neuromuscular efforts [44], with weaker individuals needing a greater level of effort to move their bodies compared with stronger individuals. Following this rationale, individuals must be strong enough to carry their body mass, and lower body fat levels would conceivably make this task easier. On these lines, high body fat levels often lead to increased metabolic demands, as the body must move and support a larger inert load during physical activities, potentially leading to the early onset of fatigue and reduced exercise tolerance [8].

In the current investigation, total lean mass, body fat percentage, Timed up-and-go time, and unloaded and loaded Stair-climbing time improved during the power training intervention. The findings correspond with previous research, indicating that power training enhances total lean mass and improves physical function in older men [20,21,22,23,28].

To the authors’ knowledge, no study to date has investigated the association between power training-induced changes in body composition and physical function in older adults. In the present study, a similar non-linear relationship (as for the baseline results) between body composition and physical function was discovered when analysing the power training-induced changes. A notable pattern emerged, wherein an impaired improvement in physical function was primarily observed in participants with a decrease in body fat percentage (⪅−1.5% body fat) but with an indication of impaired improvement in physical function in participants with an increase in body fat percentage (⪆2% body fat). On average, the participants who either preserved their body fat percentage or experienced minor alterations appeared to obtain the greatest improvements in physical function pre- to post-intervention. The observed variation underscores the inter-individual differences in how body composition changes influence physical function, highlighting that the responses to these changes can be highly individualistic. Based on previous research [45,46], a greater improvement in physical function for the participants with a reduction in body fat percentage was anticipated. However, considering the low number of obese participants (*n* = 2) and the relatively high functional ability level at baseline, the results may not be surprising. In obese older adults, it was shown that weight loss improves physical function and ameliorates frailty [46]. However, 37.5% of the participant sample in the present study was defined as having a healthy weight at baseline, and only 4.2% were defined as obese. Therefore, it can be argued that the participant sample in the present study would not have the same improvements in physical function after a reduced body fat percentage. Accordingly, a decreased body fat percentage would presumably have been more advantageous for physical function in an obese participant sample with a low functional ability level.

Furthermore, potential confounding factors, such as nutritional deficiencies and loss of muscle mass, could additionally have affected the outcomes. A rapid decrease in body fat could be associated with inadequate nutrition. Inadequate intake of essential nutrients may lead to frailty [47] and fatigue [48], which can, in turn, impair physical function. However, in the present study, we did not have the resources to conduct a comprehensive assessment of participants’ nutritional intake. Moreover, weight loss in older adults is commonly accompanied by a reduction in lean mass even while performing resistance exercises [49]. Hence, another explanation for an impaired improvement in physical function could be a co-occurring reduction in total lean mass in individuals with weight loss. However, a reduction in lean mass or weight loss was not observed in the total participant sample in the present study pre- to post-intervention. Moreover, when performing sub-analyses of the tertile that lost the most weight, a significant reduction in lean mass could still not be found. As such, a co-occurring reduction in total lean mass did probably not occur and seemed not to be the reason for an impaired improvement in physical function in the participants with a decrease in body fat percentage. Nevertheless, the present study did not have enough participants to investigate this in the tertile that lost the most weight (*n* = 16). The lack of representation of underweight and obese individuals means that the results predominantly reflect the outcomes of those within the normal to overweight range. Consequently, this might affect the generalisability of the findings, especially when considering populations with a significant proportion of underweight individuals. Future studies with more diverse body composition cohorts are needed to validate further and broaden the applicability of the present study’s findings.

### Strengths and Limitations

The present study was conducted as a pre- to post-experiment, including a cohort of older men who had high compliance with the training sessions. Experienced coaches supervised all training sessions with close follow-up during each session. Furthermore, objective measures of physical function and dual-energy X-ray absorptiometry for body composition were used, which all had relatively high levels of reliability and validity. On the other hand, the present study was a pre-test–post-test experiment and did not include a non-exercising control group. Hence, several other factors could have confounded the changes in body composition and physical function (e.g., habitual physical activity, sleep, fatigue, depression, self-esteem, energy consumption, and protein intake) [6,50,51,52].

At last, the present study had a relatively short exercise intervention duration with small pre- to post-changes. The CV for the accuracy of DXA in measuring body fat percentage and lean mass is ~2–3% [36], meaning that the changes observed in this study are within the margin of error and should be interpreted with caution. An extended intervention could have discovered more apparent body composition and physical function changes. However, the intervention was sufficient to reveal significant changes in body composition and physical function pre- to post-intervention.

Randomised controlled trials with larger sample sizes, more extended intervention periods, control of energy intake, and exercising and non-exercising control groups are needed to examine how power training-induced changes in body composition may influence improvements in physical function in older adults. Additionally, future research should delve deeper by targeting specific age groups within the older adult category to distinguish nuanced differences. It would also be valuable to consider sex differences, as hormonal and physiological variations between men and women might influence the outcomes of power training interventions and the association between body composition and physical function.

## 5. Conclusions

The present study’s findings indicate that total lean mass is positively associated with Sit-to-stand power. Additionally, total body fat and body fat percentage had a non-linear relationship with physical function in older men at baseline. The participants with a body composition of ~20% body fat displayed the highest level of physical function. Furthermore, power training-induced changes in body fat percentage had a non-linear relationship with changes in physical function. On average, despite small pre–post changes in body fat, participants who either preserved their body fat percentage or experienced minor alterations appeared to obtain the greatest improvements in physical function pre- to post-intervention. Neither excessive nor too-low body fat levels may seem beneficial for older men’s physical function.

### Practical Implications

The growing number of older adults combined with the rise in the prevalence of obesity is expected to result in higher functional limitations and physical disability levels in older adults. From a public health perspective, this could be one of the most considerable challenges of this century due to the increased burden on the healthcare system. Therefore, encouraging the older population to be more physically active with a particular focus on power training and maintaining a healthy body composition is of great importance and can potentially decelerate the age-related decline in physical function in the fast-growing older population. In line with these arguments, the observations of a potential non-linear relationship between body composition and physical function could guide interventions or policy formulations, especially in establishing an optimal body fat percentage threshold to optimise physical function in older men.

## Figures and Tables

**Table 1:** Participants’ characteristics, BMI, and results for physical function measures.

Variables	Pre	Post	Change
	Mean ± SD	Mean ± SD	Δ% ± SD
Age (yrs)	67.7 ± 5.3		
Height (cm)	178.9 ± 7.0		
Total mass (kg)	83.4 ± 10.4	83.7 ± 10.4	0.5 ± 2.5
Total body fat (kg)	22.1 ± 7.4	21.7 ± 6.8	−0.7 ± 7.3
Body fat percentage (%)	26.0 ± 6.4	25.5 ± 5.8	−1.2 ± 5.7 *
Total lean mass (kg)	57.9 ± 5.4	58.7 ± 5.7	1.3 ± 2.0 *
BMI			
Underweight (n)	0	0	
Healthy weight (n)	18	17	
Overweight (n)	28	29	
Obese (n)	2	2	
Physical function			
Sit-to-stand power (W)	1742 ± 333	1731 ± 335	−0.3 ± 7.7
Timed-up-and-go (s)	4.13 ± 0.30	4.06 ± 0.27	−1.58 ± 4.90 *
Stair climb (s)	3.74 ± 0.48	3.53 ± 0.39	−5.16 ± 6.66 *
Loaded Stair climb (s)	3.93 ± 0.44	3.76 ± 0.46	−4.07 ± 6.82 *

Yrs: years, cm: centimetres, kg: kilograms, *n*: number, W: watts, s: seconds, Δ%: per cent change. BMI classification is based on the WHO BMI category [38]. Underweight (<18.5 kg/m^2^), healthy weight (18.5–24.9 kg/m^2^), overweight (24.9–29.9 kg/m^2^), and obese (>30 kg/m^2^). Values are presented as mean ± SD. * *p* < 0.05, significant change from baseline.

## Data Availability

Data are available from the corresponding author upon reasonable request.

## Original Conference Paper

This article is a revised and expanded version of a conference paper entitled “Is body composition associated with physical function in older men?” [53], which was presented at the 27th Annual ECSS Congress in Sevilla/Spain, 30 August–2 September 2022.
